# Normative Data for Objective ADHD Smartphone Application in a General Population

**DOI:** 10.2174/0117450179377329250131072308

**Published:** 2025-02-03

**Authors:** Urban Gustafsson, Robert Nolen, Nuria Casals, Simon Larsson, Ragini Sanyal, Mikkel Hansen

**Affiliations:** 1 Qbtech AB, Medical Department, Cardellgatan 1, Stockholm, Sweden; 2 Qbtech, Inc., 8 Greenway Plaza, Suite 750, Houston, TX77046, United States

**Keywords:** ADHD, QbMobile, Normative, Objective measure, Psychometric test, Socioeconomic

## Abstract

**Background:**

Normative data for objective measures in the clinical assessment of Attention Deficit Hyperactivity Disorder (ADHD) are crucial for enhancing the accuracy, reliability, and clinical utility of diagnostic tools. This observational study aimed to establish normative demographic data in a representative population for an investigational version of QbMobile (QbMobile), a software application for the administration of a performance test to provide objective measurements of hyperactivity, inattention, and impulsivity in the clinical assessment of ADHD.

**Methods:**

This study was conducted in the United Kingdom, The Netherlands, Germany, and the United States. Participants between 6-60 years were included. The QbMobile application was pre-installed on the smart device/iPhone. Participants were instructed to sit holding the smartphone with both hands and tap the screen each time a target stimulus appeared on-screen and withhold tapping to all other stimuli. The smartphone tracked the participants' movements with the camera and movements from the smartphone to measure activity.

**Results:**

A total of 2541 participants completed the study, n=326 aged 6-11 years and n=2215 aged 12-60 years. There were more females (55.7%) than males (44.3%). Demographic and characteristics variables had no impact on the data collected. Household income and educational levels were investigated to ensure sufficient spread across countries. The basic parameters of QbMobile were captured and evaluated during the assessment. There was a high degree of acceptance of the test, and 94% reported that the application was easy to use.

**Conclusion:**

Representative normative data for QbMobile was established from a multinational general population and should provide a useful basis as a comparator for other datasets, such as people with ADHD.

## 1 INTRODUCTION

Attention deficit hyperactivity disorder (ADHD) is a common neurodevelopmental disorder typically diagnosed in childhood, affecting approximately 5% of children and adolescents and 2.5% of adults globally [1-4]. ADHD is typically diagnosed in early childhood, and the disorder is one of the most common diagnoses in educational and children’s mental health conditions [5]. Objective psychometric test quantification and classification of symptoms have shown to be a valuable decision-making tool to support clinicians during assessment and treatment monitoring as part of ADHD management [3, 6-8].

QbTest is a non-invasive computer-based test that objectively measures cognitive performance and activity levels [3, 7, 9-11]. The test provides data for the core signs of ADHD, such as attention, hyperactivity, and impulsivity. The intended use of QbTest is to provide healthcare professionals with objective measurements of hyperactivity, impulsivity, and inattention to aid in the clinical assessment of ADHD and in the evaluation of treatment interventions in these patients [3, 7, 12]. QbMobile is a software application for the administration of QbTest on a smartphone. QbMobile is pre-installed on an iPhone mobile device which includes a short video clip with test instructions that each participant watches, followed by a short practice test, before starting the full assessment. Participants’ responses are recorded *via* touching the screen of the smartphone during the test, while the camera on the smartphone tracks the movement-related activity. Furthermore, telehealth and digital technologies have become more prominent in the last several years to enhance access and support diagnostic strategies [13, 14]. Developing a mobile management system is expected to improve medication regimen adherence and facilitate communication with healthcare providers [15], and the development of mobile technology in the management of chronic diseases should also be of interest [16-18].

In order to evaluate the performance of any objective measures in neuropsychological testing it is important to obtain normative data from a representative general population [19]. Normative data (norms) is usually obtained by administering the test to a representative sample to establish norms including, for example, sociodemographic characteristics, such as age, sex, and educational level [19]. Norms are estimations that are characteristic of a group and serve as baseline metrics for comparing patient performance. A larger sample may be preferred to adequately represent the population for which the psychometric test is intended [20]. By gathering normative data, various levels of test performance can be determined and compared to the test performance of patients with the disorder in question [19-21].

The aim of this observational study was to establish normative data for QbMobile in a representative general population sample across Europe and the United States in participants aged 6-60 years. The data presented herein is based on a general populace, and the establishment of these norms will allow clinicians to assess a patient’s performance relative to an appropriate reference group.

## 2 MATERIALS AND METHODS

### 2.1 Study Conduct, Setting, and Participants

The observational study was conducted according to the ethical principles of the Declaration of Helsinki, Clinical Investigation of Medical Devices for Human Subjects/Good Clinical Practice, and any regional or national regulations, as appropriate. A CRO (Clinical Research Organization) was utilized to manage the project and execute and ensure adherence to study submissions, requirements, and ethical standards. Prior approvals of the study protocol were received from a central institutional review board (UserWise Institutional Review Board Board, The Alameda, San Jose, California, United States; QB22-01_08-11-2022). Informed consent was obtained from all participants prior to study participation.

The inclusion criteria were as follows: (a) Aged ≥ 6 years and ≤ 60 years, (b) have no documented or suspected current or lifetime diagnosis of ADHD or have a diagnosis of ADHD but currently not receiving treatment, and (c) have adequate sensory and physical ability to complete QbMobile. The exclusion criteria were as follows: (a) received any type of treatment for ADHD in the past 30 days, (b) a concurrent medical diagnosis that could significantly affect test performance (*i.e*. brain injuries, Parkinson’s disease, current epilepsy or active seizures, amyotrophic lateral sclerosis, multiple sclerosis, dementias (*e.g*., vascular dementia, Alzheimer’s disease), unmanaged psychiatric illness, and (c) substance use (*e.g*., alcohol, drugs) that may affect performance on the day of the test.

Participants were recruited through each research facility's general population and targeted ADHD database. Participants were screened *via* the online questionnaire to capture ADHD diagnosis status, technology usage, and general demographic information.

Data were collected across three European countries (the United Kingdom, The Netherlands, and Germany) and the United States, between September 2022 and July 2023. Research sites were selected to be as geographically distributed throughout cities in Europe and the US as possible (37 sites total). Strategies and resources were utilized to recruit throughout major regions to capture subpopulations (ethnic minorities, income levels, educational background, *etc*.) by adding cities where they were more likely to find and test individuals that represented the norm population. Participants were recruited across geographically diverse sites in the United States and Europe to ensure representative sampling. The screening process gathered information about age, sex, marital status, race, ethnicity, education, income, medical problems in the past, smoking habits, and alcohol and drug use. This process would ensure the study includes a robust normative population. Furthermore, three different cohorts were collected: one cohort of normative data when tested on an iPhone, one cohort to claim a subset of ADHD, and one cohort was used to collect normative data when tested on an iPad. For the purposes of this study, only the iPhone normative data is presented in this article.

### 2.2 QbMobile Test

QbMobile is a software application for the administration of a performance test identical to the QbTest, which is an objective measure that utilizes a high-resolution motion tracking system linked with a computerized Go/No-Go paradigm for children. The Go/No-Go paradigm is based on a task in which participants must press a handheld responder button each time a circle appears on-screen but withhold the response when a cross appears in front of the circle. Adolescents and adults complete a one-back task that involves four types of stimuli where the target stimulus is defined as the stimulus that is identical in shape and color to the one immediately preceding the stimulus [3, 7, 12]. QbMobile was pre-installed on the smart device/iPhone, which included a short video clip with test instructions that each participant watched, followed by a short practice test before starting the full test. The responses of the participants were recorded *via* touch screen responses on the smartphone during the test, and participants who used a stationary, mobile device responded using the spacebar on a wireless keyboard. Simultaneously, activity was measured by movement data captured from the built-in camera, as well as movement from holding the phone. The head movements of the participants were captured during the test by the ARkit technology from the smartphone. The QbMobile test duration for 6-11 years of age and 12-60 years of age was 10 minutes.

Approximately half of the eligible participants were invited to complete QbMobile at a dedicated research facility. iPhone mobile devices were provided to each participating clinic for data collection of the study. The devices were used for QbMobile Client administration only. All other eligible participants were asked to complete QbMobile remotely (at home) using their personal iPhone device that supports the QbMobile app. The QbMobile test instructions were the same for both groups and required remote participants to establish an undisturbed environment like those tested at the research facilities.

Several quality control criteria were developed after data collection to identify those tests deemed unacceptable and therefore excluded, such as the test taker must reach the end of the test. Additionally, tests must be conducted in a quiet environment to avoid loud noises. For that, the average recorded background sound level needed to be below a certain threshold. Any test performed without following the instructions was to be discarded. For instance, if the accelerometer feature from the mobile device indicated that the phone was left still or placed facing down on a table, that test would be excluded. This would be against test specifications since the users were instructed to hold the device in both hands. Tests where the test taker was not sitting in their seat or walking around during the test were discarded. Any test with prolonged response inactivity and/or the iPhone camera was unable to detect and track the participant’s face for a period of time was also excluded.

### 2.3 Data Collection

The data collection was performed from the electronic Case Report Form and from the smartphone application. The device data was collected using a custom-made iOS app. The tool used for collecting face-tracking data was ARKit [22]. Data obtained from the participants during the screening process included, but was not limited to, inclusion/exclusion criteria, ADHD diagnosis status, technology usage, education, and income levels. When participants logged into the QbMobile app, demographic and characteristic data, such as age, sex at birth, race, ethnicity, eye color, and vision, were entered and collected. Eye characteristics were collected to be able to evaluate if the face-tracking ARKit was impacted by eye wear and eye color. After the completion of QbMobile, participants were encouraged to complete a satisfaction survey to describe, rate, and offer suggestions regarding their QbMobile experience. The survey questions touched on the participant’s testing environment, test instructions, technical issues, and overall use of QbMobile. It was considered that the questionnaires were valid and reliable for the aim of the study.

The QbMobile data were processed in the QbTest software client to calculate the above parameters and then submitted and stored at a central server hosted by Amazon Web Services (AWS, Ohio). The transfer of test data between the software client and the server was encrypted and data analyses were conducted on pseudonymized data only. The data is presented as descriptive statistics with N, %, mean, or standard deviation. The study size was based on a powerful sample size (>2000) [20].

The data collected from QbMobile is used to generate test results based on several key parameters, which originated from the QbTest [11, 23]. For activity, the parameters include micro events and distance. Impulsivity was assessed using measures of commission errors, error rate, and anticipatory responses. Attention was evaluated through omission errors and reaction time. These parameters provide measurable values and scores that form the basis of the test results.

## 3 RESULTS

### 3.1 Demographics and Characteristics

The study comprised of 3920 participants. After excluding datasets due to the implemented quality controls (n=699), the iPad cohort (n=426), and the group of self-reported ADHD diagnoses-(n=254), the final normative dataset for QbMobile consisted of 2541 participants aged 6-60 years. Participants were enrolled across three European countries (the United Kingdom, The Netherlands, and Germany) and the United States (Table **1**).

Demographics (sex, age, race, and ethnicity) and characteristics (vision and eye color) are presented in Table **2**. There were 325 participants in the 6–11-year age group and 2215 participants in the 12-60-year age group. Overall, there were more females (55.7%) than males (44.3%); 58.4% were White, followed by 18.1% Black,

10.1% Asian, and 13.4% Other. Approximately 2/3 of the total population had normal vision, while 25% wore glasses (Table **2**). Demographic descriptive variables (sex at birth, age, race, ethnicity) and characteristics (vision, eye color) were similar between groups, and each country was well represented. Descriptive analysis of race, vision, and eye color did not impact the face tracking ARKit technology from the smartphone.

### 3.2 Income and Education Levels

For a subset of the sample, household income and educational levels reported by the participants were analyzed to ensure a sufficient spread, and income level was then compared to national averages. Figs. (**1** and **2**) show an adequate range and relative normal distribution of both household income and educational levels in the norm cohort in the United States and Germany together. As a reference, the average income in the United States in the year 2023 was €74 028 ($80 115), and in Germany was €60 867 ($65 816) [24].

### 3.3 Participant Survey

A subset of participants (n=484) completed a short survey after completing the smartphone task. There was a high degree of acceptance of the test, and 93.6% thought that the app was easy to use (Table **3**). The participants also answered questions about technical issues and distractions.

### 3.4 QbMobile Parameters

The mean and standard deviation (SD) of the basic parameters from QbMobile performance are given in Table **4**. The QbTest parameters are also shown in order to compare the output from both QbTest and QbMobile (the QbTest data is derived from Qbtech AB ([25]) (Table **4**).

#### 3.4.1 Error Rate

Error rate is the frequency of incorrect responses by the patient (*i.e*., the participants pressed the response button for non-targets and/or did not press for Targets).

#### 3.4.2 Commission Errors

Commission errors occur when a response is registered when the stimulus was a non-target (*i.e*., the screen is pressed when it should not have been).

#### 3.4.3 Omission Errors

Omission errors occur when no response is registered to a target stimulus (*i.e*., the screen was not pressed when it should have been).

#### 3.4.4 Anticipatory Errors

Anticipatory errors occur when a response is registered a little before or just after a stimulus is presented, *i.e*., the responder button is pressed as a result of anticipation rather than as a response to the stimulus.

#### 3.4.5 Distance

Distance is an activity parameter that measures the distance travelled by the face during the test. It is measured in meters.

#### 3.4.6 Micro Events

Micro events occur when the head changes its position more than one millimeter since the last Face microevent. Movements of less than one millimeter are not registered as a face microevent.

#### 3.4.7 Number Block

Number block is the area surface covered by the face during the test. It reflects how vivid the movements were during the test.

#### 3.4.8 Reaction Time

Reaction time is the time it takes for the test taker to press the screen after a stimulus has been presented. Reaction Time Mean is the average time it takes for the patient to press the response button after the stimuli have been presented. The Reaction Time Mean is measured only when a correct response is registered.

Overall, the parameters from QbMobile were found to be comparable to the output parameters of the normative data from QbTest.A similar pattern was observed for the variables with respect to sex and age between the two tools. This further confirms the validity of the normative data for the QbMobile.

## 4 DISCUSSION

For this QbMobile investigation, a large sample size of 2541 participants was collected for normative data control, representing a multinational, general population across Europe and the United States. Based on the reported educational level and household income, the cohort was sufficiently diversified, and income levels matched the national average in the participants’ respective countries. The age range was between 6-60 years, with somewhat more females (55.7%) than males (44.3%) overall. Demographic variables (sex, age, race, ethnicity) and characteristics (vision, eye color) had no impact on the data collected. There was a high level of acceptance of the test, with few technical errors reported during the test, which were not related to correctly implemented quality controls or post-test data upload issues. The study objective of establishing a high-quality normative cohort with a balanced representation across socio-economic classes, demographics, and different environmental settings was successfully achieved.

The aim of establishing normative data in psychometric tests is to evaluate an individual patient compared to a large group of people, analogous for their age, gender, and level of education, allowing for something to be inferred about the individual's level of impairment at a particular time point [21]. Age and education level have been stated to play crucial roles in shaping normative data for psychometric tests [26]. Accounting for these factors through appropriate stratification and adjustments could be essential for accurate test interpretation and fair assessment across diverse populations. Thus, normative data stratified by age, sex, and education may allow for more precise and accurate diagnoses, especially in cognitive and psychological conditions [27]. Furthermore, in a recent systematic review that analyzed normative data estimation in neuropsychologic testing, norm data have mainly been focused on adults and less common for children and adolescents, and most studies have developed normative data for a single country rather than several countries simultaneously [19]. The intention is that the results, for example, from a neuropsychological test, will seek to improve the objective quantification and accuracy of the classification of symptoms, as seen in people with ADHD, or similar to test takers whose age and sex matched with the normative controls.

The size of the normative sample should be large enough to adequately represent the population and its various demographic subgroups [28, 29]. It is generally considered that a sample size of n= 50-100 could be sufficient to obtain stable means for normative test data [29, 30]. Certainly, larger sample sizes are preferred because this tends to provide more accurate and stable normative data [20]. One of the first continuous performance tests, called the Gordon Diagnostic System, comprised of normative data based on 1266 children (aged 4-16 years from the United States) [31]. The use of normative data for the Test of Attention of Analysis was based on 775 children (aged 6-16 years, population from the United States) [32], and the Conners Continuous Performance Test consisted of 816 children (aged 9-17 years, population from United States) [33]. QbTest has a similar amount of normative data (N=1307) for subjects in the age range of 6 to 60 years (n=576 for ages 6-12 years and n=731 for 12-60 years) (population from Sweden and Germany) [25]. Taking this into account, the normative data number of participants of 2541 for QbMobile is considered as a robust sample size [20].

Socioeconomic status is a complex and manifold concept that involves several components, such as income, education, occupation, and wealth [34]. The spreading of socioeconomic status varies significantly across countries and regions, reflecting disparities in economic development, social structures, and policy frameworks [35, 36]. A normal distribution was found in the present study based on education and household income depiction of the normative data. While the normal distribution is an appropriate model, it could be noted that socioeconomic breakdown could be influenced by various factors, such as income inequality, education disparities, and social mobility, which could lead to skewed distributions [37]. Socioeconomic disadvantage correlates with the risk of ADHD [38], and this could be of importance to address in the evaluation between normative and the disorder in question [39-41].

The error rate, commission rate, and omission rate parameters from QbMobile are the same measurements as the QbTest parameters. ObMobile extends QbTest functionality by incorporating 3D motion tracking, enhancing precision in activity measurements. All the face tracking parameters, Time active, Total distance, and Micro events are tracked in 2D (two-dimensional) for QbTest but are tracked in 3D (three-dimensional) for QbMobile. Similarly, the area in QbTest is 2D, but the number of blocks in QbMobile are 2D and 3D equivalents of each other. Another difference is that QbMobile’s camera is not in a truly static position and some face movement might be due to hand movements. Reaction-based parameters might see a difference because of differences in the test setup. In QbTest, a mechanical actuator is being held, while QbMobile requires a screen tap, which might take longer or shorter to perform.

There are some limitations of the study to be considered. The study was performed in the United Kingdom, The Netherlands, Germany, and the United States, and further countries included would have been preferable. Only considering the geographical Western world could be a limitation, and the normative sample established in our study may not precisely represent the broader population globally, which could imply biased or imprecise norms [42]. Besides, normative data collected in one geographic area may thus not be representative of populations in other regions due to cultural, socioeconomic, or environmental differences [43]. Geographic normative data often focus on traditional demographic variables like age, race, ethnicity, sex, and education while overlooking other important factors, such as economic status, language, neighborhood conditions, or migration status [21]. Normative data may also become outdated over time due to population-level changes in performance [21].

## CONCLUSION

Normative data for the objective psychometric measure of QbMobile in a smartphone application was established with a robust sample size from a general multinational population (age range 6-60 years). Demographic and characteristic variables had no impact on the data collected. The majority of the test takers reported that the application was easy to use, with a high degree of acceptance of the test. The basic parameters of QbMobile were captured and evaluated during the assessment and were found to be comparable to the normative data of QbTest as well. Moreover, the normative data from QbMobile should provide a useful subset framework in the clinical settings, for instance, when QbMobile is administered as an added diagnostic tool in the clinical assessment of ADHD and the evaluation of treatment interventions.

## Figures and Tables

**Fig. (1):**
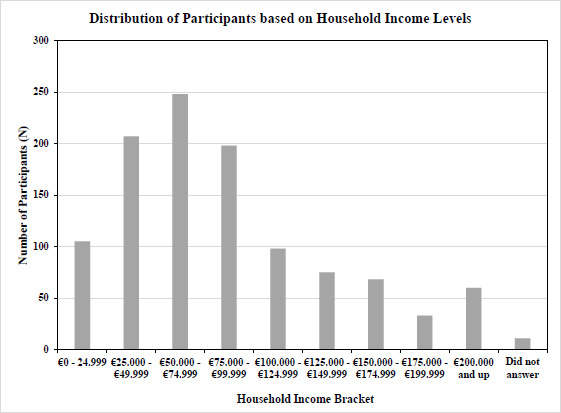
Distribution of reported household income in a subset of participants (Germany and the United States together) (income brackets in Euro on the X-axis, number of subjects on Y-axis, N=1103).

**Fig. (2):**
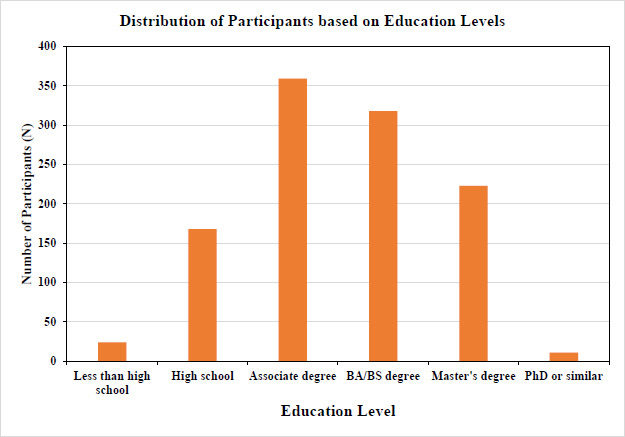
Distribution of education level (Germany and the United States together). Educational level is represented as a scale from 0-5; 0 = Less than high school level, 1=High school, 2= Associate degree, 3= BA/BS degree, 4=Master’s degree, and 5 = Ph.D. or similar (educational level on X-axis and the number of subjects on Y-axis, N=1103).

**Table 1:** Participants by country (number of participants).

**Country**	**N (%)**
United Kingdom	1052 (41.4%)
The Netherlands	357 (14.0%)
Germany	392 (15.5%)
United States	740 (29.1%)
Total	2541 (100%)

**Table 2:** Demographics and characteristics by age group (number of participants).

***Demographics***	**6–11-year Age Group** **n=326**	**12–60-year Age Group** **n=2215**	**6-60-year Age Group (Total)** **N=2541**
**Age (Years)**	-	-	-
Mean	8.7	31.6	28.7
SD	1.8	13.4	14.7
Min	6	12	6
Max	11	60	60
**Sex**	-	-	-
Male	153 (46.9%)	972 (43.9%)	1125 (44.3%)
Female	173 (53.1%)	1243 (56.1%)	1416 (55.7%)
**Race**	-	-	-
White	157 (48.1%)	1326 (59.9%)	1483 (58.4%)
Black	82 (25.2%)	379 (17.1%)	461 (18.1%)
Asian	32 (9.8%)	225 (10.2%)	257 (10.1%)
Other	55 (16.9%)	285 (12.9%)	340 (13.4%)
**Ethnicity**	-	-	-
Not Hispanic or Latino/a, or Spanish origin	240 (73.6%)	1752 (79.1%)	1992 (78.4%)
Hispanic, or Latino/a, or Spanish origin	34 (10.4%)	162 (7.3%)	196 (7.7%)
Prefer not to say	13 (4.0%)	65 (2.9%)	78 (3.1%)
Unknown	39 (12.0%)	236 (10.7%)	275 (10.1%)
	-	-	-
***Characteristics***	**n=326**	**n=2215**	**N=2541**
**Vision**	-	-	-
Normal (no correction or vision supports used during test)	276 (84.7%)	1373 (62.0%)	1649 (64.9%)
Glasses	49 (15.0%)	604 (27.3%)	653 (25.7%)
Contact Lenses	1 (0.3%)	213 (9.6%)	214 (8.4%)
Other	0	25 (1.1%)	25 (1.0%)
**Eye Color**	-	-	-
Brown	221 (67.8%)	1254 (56.6%)	1475 (58.0%)
Blue	65 (19.9%)	505 (22.8%)	570 (22.4%)
Green	28 (8.6%)	325 (14.7%)	353 (13.9%)
Hazel	7 (2.2%)	49 (2.2%)	56 (2.2%)
Black	1 (0.3%)	18 (0.8%)	19 (0.8%)
Other	4 (1.2%)	65 (2.9%)	68 (2.7%)

**Table 3:** Participants’ survey results (N, %).

**Query**	**Strongly Agree n (%)**	**Agree** **n (%)**	**Disagree** **n (%)**	**Strongly Disagree** **n (%)**	**Total N (%)**
The instructions on how to take the test were easy to understand.	236 (48.7%)	189 (39.0%)	38 (8.0%)	21 (4.3%)	484 (100%)
The mobile app was easy to initiate and follow through to completion.	242 (50.0%)	197 (40.7%)	33 (6.8%)	12 (2.5%)	484 (100%)
Overall, the mobile app was easy to use.	255 (52.8%)	197 (40.8%)	22 (4.6%)	9 (1.8%)	483 (100%)

**Table 4:** Output of basic parameters from the QbMobile test (N=2541) (upper panel). For comparison, the QbTest parameters are also shown (N=1307) (lower panel) (QbTest data were derived from standardized subsequent duration of the task, and collected from Qbtech AB ( [25]). Values are given as Mean (SD).

**QbMobile** **Parameter** **(Mean (SD))**	**All (N=2541)**	**Male** **6-11 years** **(n=153)**	**Female** **6-11 years** **(n=173)**	**Male** **12-60 years** **(n=972)**	**Female** **12-60 years** **(n=1243)**
Error rate (%)	6.2 (6.0)	13.4 (8.4)	13.1 (7.9)	5.3 (5.1)	5.1 (4.6)
Commission error (%)	4.7 (8.1)	19.4 (12.6)	17.9 (12.2)	2.9 (4.8)	2.6 (4.2)
Omission error (%)	10.1 (11.4)	7.8 (8.4)	8.1 (8.5)	10.6 (12.2)	10.3 (11.4)
Norm commission error (%)	5.5 (9.4)	21.7 (14.9)	19.9 (13.6)	3.5 (6.2)	3.0 (5.1)
Anticipatory error (%)	0.5 (1.6)	2.3 (3.4)	1.6 (2.2)	0.3 (1.5)	0.2 (1.0)
Time active (%)	16.8 (17.4)	33.8 (24.0)	31.5 (21.4)	16.6 (16.4)	13.0 (14.0)
Total distance (m)	4.8 (3.7)	7.9 (4.9)	7.7 (4.9)	4.9 (3.7)	4.0 (2.9)
Number of blocks (cm^3^)	209.6 (281.8)	397.3 (358.3)	398.5 (391.9)	213.5 (297.7)	157.2 (210)
Micro events (count)	2824.8 (2080.1)	4538.4 (2547.7)	4465.3 (2313.3)	2839.8 (2101.0)	2374.0 (1709.5)
Reaction time mean (ms)	534.8 (119.1)	433.1 (96.2)	456.7 (99.0)	535.9 (115.8)	557.3 (115.7)
Reaction time std (ms)	127.0 (43.8)	121.5 (42.5)	123.4 (40.4)	126.8 (46.2)	128.3 (42.4)
**QbTest** **Parameter** **(Mean (SD))**	**All (N=1307)**	**Male** **6-11 years** **(n=262)**	**Female** **6-11 years** **(n=314)**	**Male** **12-60 years** **(n=360)**	**Female** **12-60 years** **(n=371)**
Error rate (%)	5.9 (7.3)	12.7 (10.3)	8.2 (7.3)	2.6 (2.2)	2.5 (2.2)
Commission error (%)	6.7 (9.8)	17.2 (12.9)	10.6 (9.1)	1.5 (1.5)	1.3 (1.4)
Omission error (%)	5.9 (7.2)	6.4 (9.0)	5.2 (8.1)	6.0 (5.8)	5.9 (6.2)
Norm commission error (%)	7.4 (11.2)	19.2 (15.3)	11.5 (10.3)	1.6 (1.8)	1.4 (5.9)
Anticipatory error (%)	------	1.0 (2.0)	0.3 (1.1)	-------	-------
Time active (%)	17.9 (21.7)	38.0 (25.2)	28.8 (28.9)	6.6 (7.8)	5.5 (6.3)
Total distance (m)	5.6 (4.9)	9.4 (6.8)	7.2 (5.2)	3.7 (2.3)	3.3 (1.5)
Area (cm^2^)	26.4 (26.9)	49.5 (33.2)	38.0 (28.3)	13.8 (12.9)	12.6 (10.4)
Micro events (count)	3385.1 (2828.6)	5843.3 (3432.4)	4593.0 (2860.0)	2068.7 (1474.9)	1904.1 (1224.7)
Reaction time mean (ms)	517.2 (110.6)	466.7 (104.0)	494.4 (127.5)	539.6 (97.4)	550.2 (93.4)
Reaction time std (ms)	142.2 (50.3)	138.9 (56.7)	130.1 (53.2)	148.9 (46.8)	148.2 (44.1)

## Data Availability

The authors confirm that the data supporting the findings are available within the article. Due to the nature of the research, limited access to supporting data may be granted upon reasonable request under confidentiality agreements from the corresponding author [U.G].

## LIST OF ABBREVIATIONS

ADHD: = Attention Deficit Hyperactivity Disorder
CRO: = Clinical Research Organization

## STANDARDS OF REPORTING

STROBE guidelines were followed.
